# Tritrophic interactions between a fungal pathogen, a spider predator, and the blacklegged tick

**DOI:** 10.1002/ece3.4271

**Published:** 2018-07-13

**Authors:** Ilya R. Fischhoff, James C. Burtis, Felicia Keesing, Richard S. Ostfeld

**Affiliations:** ^1^ Cary Institute of Ecosystem Studies Millbrook New York; ^2^ Department of Natural Resources Cornell University Ithaca New York; ^3^ Bard College Annandale‐on‐Hudson New York

**Keywords:** antipredator behavior, intraguild predation, *Ixodes scapularis*, *Metarhizium brunneum*, microcosm, nonconsumptive effects, trait‐mediated effects, trophic interactions

## Abstract

The blacklegged tick *Ixodes scapularis* is the primary vector for the bacterium causing Lyme disease in eastern North America and for other medically important pathogens. This species is vulnerable to attack by fungal pathogens and arthropod predators, but the impacts of interactions between biocontrol agents have not been examined. The biocontrol agent Met52^®^, containing the entomopathogenic fungus *Metarhizium brunneum* (=*M. anisopliae*), controls blacklegged ticks with efficacy comparable to chemical acaricides. The brush‐legged wolf spider *Schizocosa ocreata* is a predator of *I. scapularis* that reduces their survival under field conditions. We conducted a field microcosm experiment to assess the compatibility of Met52 and *S. ocreata* as tick biocontrol agents. We compared the fits of alternative models in predicting survival of unfed (flat) and blood‐fed (engorged) nymphs. We found the strongest support for a model that included negative effects of Met52 and *S. ocreata* on flat nymph survival. We found evidence for interference between biocontrol agents, with Met52 reducing spider survival, but we did not find a significant interaction effect between the two agents on nymph survival. For engorged nymphs, low recovery rates resulted in low statistical power to detect possible effects of biocontrol agents. We found that nymph questing activity was lower when the spider was active above the leaf litter than when the spider was unobserved. This provides the first evidence that predation cues might affect behavior important for tick fitness and pathogen transmission. This study presents field microcosm evidence that the biopesticide Met52 and spider *Schizocosa ocreata* each reduced survival of blacklegged ticks *Ixodes scapularis*. Met52 reduced spider survival. Potential interference between Met52 and the spider should be examined at larger scales, where overlap patterns may differ. Ticks were more likely to quest when the spider was inactive, suggesting the ticks changed their behavior to reduce danger.

## 1 INTRODUCTION

Intraguild interference can strongly mediate the effects of predators on prey. For example, due to interference between wolf spiders and carabid beetles, a doubling of carabids resulted in no impact on densities of herbivore pests of squash (Snyder & Wise, 1999). Predator effects on prey are further determined by the combined effects of consumptive and nonconsumptive impacts. Nonconsumptive impacts, including changes in dispersal or foraging, can be of equal or greater ecological impact compared to consumptive effects (Mestre, Bucher, & Entling, 2014; Schmitz, Beckerman, & Brien, 1997; Steffan & Snyder, 2010). For example, chemotactile residues from the prior presence of spiders (*Pisaura mirabilis*) on enclosed plants (*Urtica dioica*) reduced arthropod damage to leaves by 50% when compared with control plants (Bucher, Menzel, & Entling, 2015). Research on intraguild predation has focused on effects on herbivores (Prasad & Snyder, 2004; Saito & Brownbridge, 2016) and carnivores (Sitvarin & Rypstra, 2014). Fewer studies have examined the effects of intraguild predation on prey that are disease vectors (Caillouët, Carlson, Wesson, & Jordan, 2008). Research on nonconsumptive effects in vectors has focused on mosquitoes (Vonesh & Blaustein, 2010). In *Anopheles coluzzii*, for example, exposure of larvae to the predatory backswimmer *Anisops jaczewskii* caused negative effects on life history traits that might reduce malaria transmission (Roux et al., 2015).

Because of their importance as vectors of pathogens affecting humans, livestock, and wildlife, ticks have been the subject of extensive research on biocontrol. The efficacy of various biocontrol agents has been examined, including nematodes (Hartelt et al., 2008), bacteria (Zhioua, Heyer, Browning, Ginsberg, & Lebrun, 1999), parasitic wasps (Stafford, Denicola, & Kilpatrick, 2003), arthropod predators (Burtis & Pflueger, 2017; Samish, Gindin, Alekseev, & Glazer, 2001), and fungi (Bharadwaj & Stafford, 2010). Diverse arthropod predators have been found to prey upon ticks (Burtis & Pflueger, 2017; Samish & Alekseev, 2001). In microcosms, overwinter survival of *I. scapularis* nymphs was negatively correlated with increased abundance of large (>1 mm) arthropod predators and with predator family richness (Burtis, Ostfeld, Yavitt, & Fahey, 2015). Addition of brush‐legged wolf spiders *Schizocosa ocreata* (Araneae: Lycosidae) to soil core microcosms reduced survival of unfed (flat) *I. scapularis* nymphs by 33% (Burtis & Pflueger, 2017).

Among tick biocontrol agents, entomopathogenic fungi have demonstrated the greatest potential. Field application of Mexican strains of *Metarhizium anisopliae* reduced *Rhipicephalus microplus* larvae by 37%–94% (Alonso‐Díaz et al., 2007). *Metarhizium brunneum* strain F52, previously classified as a strain of *M. anisopliae* (Bischoff, Rehner, & Humber, 2009), has been incorporated into Met52^®^ (Novozymes Biological, Franklinton, NC, USA). Field tests with Met52 resulted in reductions in *I. scapularis* comparable to those achieved with bifenthrin, a synthetic pyrethroid (Bharadwaj & Stafford, 2010).

The Tick Project (http://www.tickproject.org) is a 5‐year study (2016–2020) to determine whether controlling ticks at the neighborhood scale reduces tickborne disease. The Tick Project is evaluating two methods of tick control: (a) Met52 and (b) bait boxes that apply the acaricide fipronil to small mammals. These two interventions were selected based on their commercial availability, efficacy, and safety. Given continued increases in tickborne diseases (Nelson et al., 2015) and public concerns about chemical control agents (Aenishaenslin et al., 2017), Met52 has the potential to be used at increasing scales.

A full assessment of Met52 must evaluate not only its efficacy in reducing tickborne disease risk in people, but also its impacts on nontarget organisms. In a Before‐After‐Control‐Impact study, we found that use of Met52 for tick control in residential yards is unlikely to cause meaningful reductions in the abundance of nontarget arthropods (Fischhoff et al. 2017). That study considered the nontarget arthropod community as a whole, and measured effects at the level of taxonomic order.

The efficacy of Met52 against ticks could be reduced if Met52 interferes with native predators of ticks. Studies using strains of *Metarhizium anisopliae* have found no effects on survival of wolf spiders (Araneae: Lycosidae) (Thang. & Shepard., 1988) in the laboratory or on abundance of wolf spiders in the field (Peng, Wang, Yin, Zeng, & Xia, 2008). Exposure to the *M. brunneum* F52 or BIPESCO 5 (=F52) strain caused increased mortality in the predatory bug *Orius majusculus* (European Commission, 2008) but not in lacewings *Chrysoperla carnea* (U.S. Environmental Protection Agency, 2000). Met52 also caused increased mortality in predatory rove beetles *Dalotia coriaria* and mites *Stratiolaelaps scimitus* and *Gaeolaelaps gillespiei* used to control Western flower thrips *Frankliniella occidentalis* (Saito & Brownbridge, 2016). These predators and Met52 were nonetheless compatible biocontrol agents: the combination of Met52 and predators suppressed thrips to a greater degree than either predators or Met52 alone (Saito & Brownbridge, 2016).

In our study, we consider the fungus and the wolf spider to be within the same ecological guild for convenience of terminology and to recognize that they exploit similar resources as generalists that feed on a wide range of arthropods (Simberloff & Dayan, 2008; European Commission 2014; Wagner and Wise 1996). We consider any spider mortality caused by the fungus to be intraguild predation, using the definition of intraguild predation as the killing and eating of species that use similar resources (Polis, Myers, & Holt, 1989). We consider any reduction in tick control due to interaction between fungus and spider to be intraguild interference (Lang 2003). The potential for intraguild predation between fungal entomopathogen and predator is asymmetric: a pathogen may infect a predator, but not the reverse (Meyling & Hajek, 2010). The concept of intraguild predation has been applied widely in biological control, for example identifying frequent infection of pathogens in both herbivore pests and parasitoids of the herbivores (Rosenheim, Kaya, Ehler, Marois, & Jaffee, 1995). Models demonstrate that the conditions for coexistence of two consumers of a common resource hold equally for systems of predator–prey, host–parasitoid, and host–pathogen communities (Borer, Briggs, & Holt, 2007).

Both wolf spiders and entomopathogenic fungi may have a combination of consumptive and nonconsumptive effects on ticks. We were particularly interested in effects of Met52 or wolf spiders on tick questing activity, as this behavior strongly affects tick‐human contact rates and therefore disease transmission (Randolph, 2004; Schulze, Jordan, & Hung, 2001). Behavioral avoidance of *Metarhizium* has been observed in Japanese beetles (Coleoptera: Scarabaeidae) (Villani et al., 1994) and Hemipteran predators (Pourian, Talaei‐Hassanloui, Kosari, & Ashouri, 2011). Spiders have nonconsumptive effects on prey (Bucher, Binz, Menzel, & Entling, 2014a; Rendon, Whitehouse, & Taylor, 2016). Certain arthropod species increase foraging and activity in response to chemotactile cues of wolf spiders (Bucher, Binz, Menzel, & Entling, 2014b; Rendon et al., 2016), while other species decrease activity in response to these cues (Bucher et al., 2014a). In a meta‐analysis, cues from predators with sit‐and‐pursue hunting styles, such as wolf spiders, caused stronger effects on prey activity, growth, reproduction, and survival, compared to effects of cues from predators with sit‐and‐wait or active pursuit hunting styles (Preisser, Orrock, & Schmitz, 2012).

**Figure 1:**
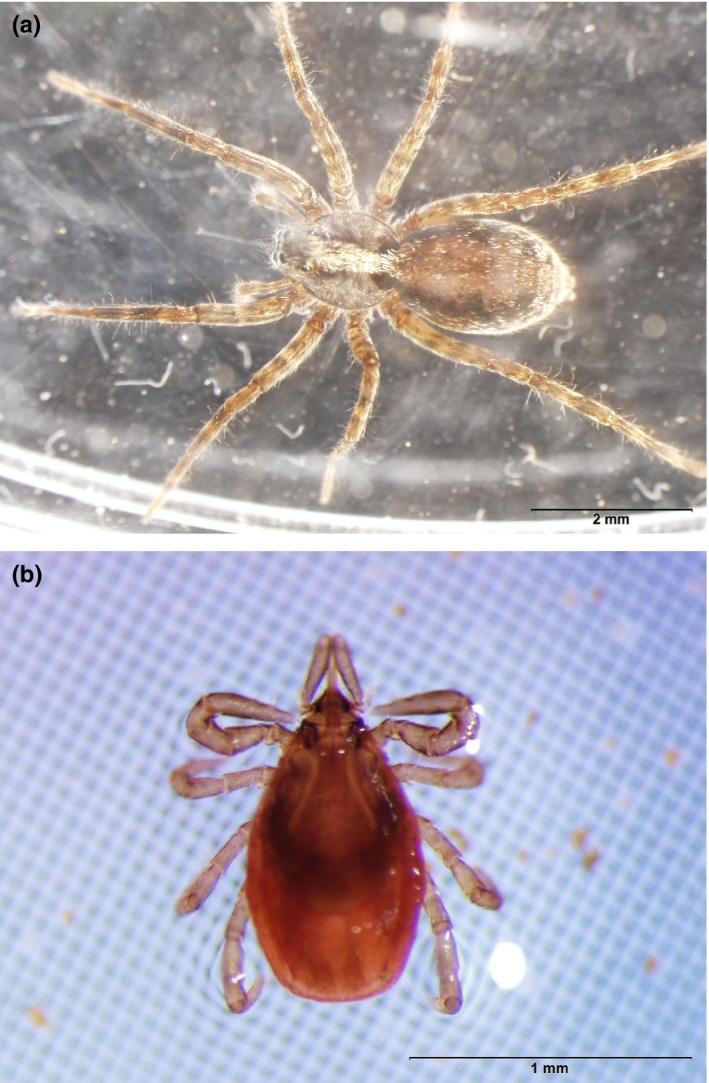
Photograph of (a) *S. ocreata* and (b) flat (unfed) *I. scapularis* nymph

We assessed effects of Met52 and *S. ocreata* on survival and questing behavior of *I. scapularis* nymphs in soil core microcosms (see Figure 1 for photographs of *S. ocreata* and of a flat *I. scapularis* nymph). We used a fully crossed factorial design, including microcosms receiving a wolf spider or no wolf spider, and sprayed with Met52 or with water as a control. We predicted that the addition of either *S. ocreata* or Met52 would reduce tick survival in the microcosms. Because there was no evidence that the fungus affected spider survival, we predicted that the two interventions together would reduce tick survival most dramatically through their combined effects. We expected that Met52 and wolf spiders each had the potential for nonconsumptive effects on ticks, given the effects of *M. brunneum* and wolf spiders on behavior of other species.

## 2 MATERIALS AND METHODS

### 2.1 Field site

We established soil core microcosms in a forest plot measuring approximately 10 m by 50 m (41°48′15.8″N 73°43′44.5″W) adjacent to a dirt road on the campus of the Cary Institute of Ecosystem Studies (CIES). The dominant tree species in the plot was sugar maple (*Acer saccharum*). The understory included sugar maple saplings, Virginia creeper *Parthenocissus quinquefolia*, poison ivy *Toxicodendron radicans*,* Sassafras albidum*, and Japanese barberry *Berberis thunbergii*.

We established 88 microcosms from 21 July to 28 July 2017, including the following treatments:

1. No spider (spider control), H_2_O spray (Met52 control) (*N* = 21 microcosms)
2. Spider addition, H_2_O spray (*N* = 20)
3. No spider, Met52 spray (*N* = 24)
4. Spider addition, Met52 spray (*N* = 23)

Each microcosm contained fifteen flat *I. scapularis* nymphs and two engorged nymphs, while spider treatments contained one female spider. We placed microcosms in randomly selected locations across a 39 by 7 grid pattern, with points on the grid 1.25 m apart. Each microcosm location was well‐shaded at midday due to their position under a closed canopy. We encircled the plot with snow fencing, 120 cm in height, to reduce wildlife disturbance.

### 2.2 Spider collection

We collected *S. ocreata* from the experimental plot and adjacent forest, and from a second, nearby forest location (41°48′9.92″N; 73°44′30.18″W). All *S. ocreata* collected were female, avoiding the possible confounding effect of sexually dependent differences in feeding habit (Walker & Rypstra, 2002). We deployed similar proportions of gravid and nongravid females in Met52‐treated microcosms (12 gravid females, 11 nongravid females) vs. control (water‐sprayed) microcosms (12 gravid females, eight nongravid females). We kept spiders in humidified vials between collection in the field and addition to microcosms, to which they were added within 3 days of collection. We did not feed spiders between collection and addition to microcosms.

### 2.3 Tick collection and deployment in microcosms

We collected flat (unfed) *I. scapularis* nymphs between 3 June and 28 July 2017 from the grounds of the CIES campus by dragging a 1 m^2^ cloth, suspended from a wooden dowel, across the forest floor and understory. We collected engorged nymphs from naturally infested white‐footed mice (*Peromyscus leucopus)* and eastern chipmunks (*Tamius striatus)*. We live‐trapped these rodents using Sherman traps between 28 June to 2 July 2017 on the CIES campus (CIES Institutional Animal Care and Use Committee Protocol 2017‐02). Each rodent was brought into the CIES rearing facility, where it was housed individually in a wire mesh cage, with ad libitum food (apple slices and rodent chow) and water, for up to 4 days, prior to its release at the point of capture. We suspended the cages within white plastic bins lined at the bottom with paper towels saturated with deionized water. Every day, we checked the paper for engorged nymphs that had fed to repletion, detached, and fallen off into the bin. We stored all nymphs at room temperature in glass vials (Wheaton item number 225536, Millville, NJ, USA) containing a 0.5 cm layer of Plaster‐of‐Paris saturated with deionized water, until we added the ticks to microcosms. We examined ticks prior to adding them to cores to confirm viability.

### 2.4 Microcosm design

**Figure 2:**
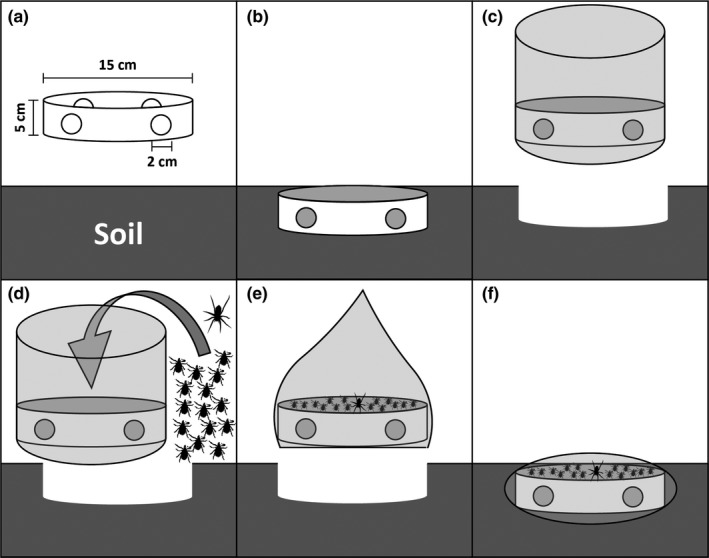
Each microcosm was contained within a section of PVC pipe (a). We dug each soil core into the soil (b) and placed it into an organza bag (c). We added 15 flat nymphs, two engorged nymphs (not shown), and one *Schizocosa ocreata*, if the microcosm was receiving a spider treatment (d). Then, we sprayed the microcosm with Met52 or H_2_O (not shown), sealed the organza bag (e), and placed the microcosm back into its original soil divot (f). Figure modified from figure 1 in Ref. (Burtis, 2017)

Each soil core microcosm was contained within a section of 15‐cm‐diameter by 5‐cm‐deep Schedule 40 PVC pipe (Brunner, Killilea, & Ostfeld, 2012) (Figure 2a). We drilled nine 1‐cm‐diameter evenly spaced holes in the walls of each PVC piece, to facilitate exchange of air and moisture between the interior and exterior of the core. We dug in each soil core by first setting a PVC piece on the ground and using pruning shears to cut around the leaf litter contained by the PVC. We temporarily set aside the leaf litter. Then we used the shears to dig a circular trench 5 cm deep and with width matching the thickness of the PVC pipe wall (0.5 cm). We pushed the PVC into the trench so that it was completely submerged in the soil (Figure 2b). The soil plug enclosed in PVC was removed with a wide spatula and placed into a tightly stitched organza bag (Figure 2c) (Quick Candles, Piedmont, SC, USA). We then replaced the leaf litter on top of the soil core and added ticks and treatments.

We used a size 0 paintbrush to move fifteen flat nymphs and two engorged nymphs from their humidified vials onto the leaf litter surface in each microcosm (Figure 2d). We distributed nymphs evenly by date of collection among microcosms in the four treatment categories. For each microcosm we had designated for spider treatment, we added one *S. ocreata*. For microcosms we had designated for fungus treatment, we then sprayed the core with 9 ml of diluted Met52. Relative to the area of the core (176 cm^2^), we used a volume of Met52 equivalent to 9 ounces (266 ml) of Met52 in 12 gallons (45.4 L) of water per 1000 square feet (93 square meters). This is three times the dosage recommended by the manufacturer and equivalent to 1.4 × 10^11^ spores/m^2^. We chose this dosage based on results from pilot experiments. *I. scapularis* were effectively controlled in yards at an even higher dose of 2.5 × 10^11^ spores/m^2^ (Stafford & Allan, 2010). For cores receiving Met52, we turned the organza bag inside out and sprayed its interior surfaces with Met52 diluted in water before we put the organza bag around the core. We sprayed the bag based on frequent observations during a pilot experiment of flat nymphs crawling on the interior of the bag. The bag received a spray volume equivalent to 3 ounces (89 ml) of Met52 concentrate in 4 gallons of water (15.1 L), per 1,000 square feet (93 square meters). This dosage is equivalent to 4.8 × 10^10^ spores/m^2^, which matches the manufacturer's recommendations (Novozymes Biologicals Inc., 2012). For microcosms receiving water (control) treatment, we sprayed the interior of the bag with an equivalent volume of water. We conducted all sprays with a hand‐pumped backpack sprayer (SOLO USA, Newport News, VA, USA), a TeeJet MeterJet spray gun (TeeJet Technologies, Glendale Heights, IL, USA), and a TeeJet TG‐3 full cone spray tip.

### 2.5 Sampling procedures

#### 2.5.1 Assessments of tick and spider activity in microcosms

On a weekly basis, we assessed tick and spider activity in the microcosms. During an activity assessment, we recorded the number of ticks visible within the core, sampling over four successive 30‐second periods. Ticks not seen during these intensive inspections, but accounted for as alive at the end of the experiment, were assumed to be immobile within the leaf litter or soil, whereas those visible above the leaf litter were considered questing for a host. Over the 2‐min period, we also recorded whether we observed the spider alive.

#### 2.5.2 Recovery of ticks and spiders from microcosms

We removed each microcosm from the field 21 days after deployment because peak reduction in *I. scapularis* nymphs has been observed 3 weeks following yard treatment with Met52 (Bharadwaj & Stafford, 2010). We stored each microcosm in a resealable plastic bag at room temperature for less than 24 hr before processing.

Following retrieval of microcosms from the field, we hand‐searched the bag, litter, soil, and PVC piece in each microcosm for 30 min in a white plastic bin. We recorded the numbers of live flat nymphs, engorged nymphs, and live adult *S. ocreata* recovered from each core. In some cases, the engorged nymphal *I. scapularis* had molted into an adult. We included engorged nymphs and molted adults in the same total for statistical analyses of treatment effects on engorged nymphs.

After hand‐searching samples, we placed the soil and litter of each microcosm into a 7.6 L Berlese funnel over a container of 70% ethanol. We wrapped each sample loosely in grade 10 cheesecloth and then placed it on top of a disk of 1.3 cm wire mesh in the funnel. We positioned a clamp light directly on top of the funnel, with a 7.5 W bulb for 1 day, followed by 15 W for 1 day, then 25 W for 2 days. If a sample remained moist after 4 days then we left it in the funnel for up to two additional days. This procedure has been found to be effective for the recovery of flat and engorged *I. scapularis* from microcosms (Burtis, 2017). After we had collected a sample from the Berlese funnel, we visually inspected the sample for 30 s under bright light and recorded any ticks or adult *S. ocreata* observed. Whenever debris inhibited thorough visual inspection, we examined the sample under a dissecting microscope. We added observations of ticks and *S. ocreata* in these samples to the values obtained from hand‐searching each microcosm. Spiders were preserved in 70% ethanol and were confirmed to be *S. ocreata* using keys (Bradley, 2012; Ubick & Cushing, 2005).

### 2.6 Statistical procedures

#### 2.6.1 Spider reproductive status

Prior to analyzing effects of *S. ocreata* on tick survival, we determined whether spider reproductive status was an important factor to include in our analysis. We fitted two alternative models for the fraction of flat nymphs recovered from microcosms at the end of the experiment, as predicted by either spider treatment without reproductive status information (spider addition vs. no spider), or spider treatment including reproductive status (gravid spider, nongravid spider, or no spider). We fitted each model to the data using the “lm” function in R. We used R version R 3.4.0 for all analyses (R Core Team, 2017). We compared the fits of the two models using the Akaike information criterion for small samples, AICc, with R package “AICcmodavg” (Anderson & Burnham, 2002; Mazerolle, 2017). We considered models with ΔAICc < 2 to have a similar level of support (Anderson & Burnham, 2002). We found similar levels of support for the model without reproductive status as for the model with this information (ΔAIC = 0.43) (Appendix S1: Table S1). Therefore, we carried out remaining analyses without specifying spider reproductive status.

#### 2.6.2 Effects of Met52 and spiders on tick survival

We constructed alternative linear models to predict the number of flat nymphs recovered at the end of the experiment, as a fraction of the nymphs originally added. These alternative models included a null (intercept‐only) model, a Met52 model, a spider model, a model including both Met52 and spider effects, and a model including effects of Met52, spider, and a spider*Met52 interaction. We compared the fits of alternative models using AICc. We computed Akaike weights based on the relative likelihood of each model: *L *= exp (−0.5*ΔAICc), where ΔAICc is the difference between each model's AICc value and the minimum AICc across models (Wagenmakers & Farrell, 2004). We applied the same statistical approach to analyzing effects of treatments on recovery of engorged nymphs.

#### 2.6.3 Effects of Met52 on spider survival

We used AICc values to compare the fit of two alternative models for spider survival: a model that included an effect of Met52 treatment and a null (intercept) model.

#### 2.6.4 Effects of Met52 and spiders on tick behavior

As a measure of tick questing activity, we used the average number of flat nymphs observed in the final activity assessment of a microcosm, as a fraction of the number of flat nymphs recovered from that microcosm. We constructed alternative models for tick activity based on all possible combinations of the following four predictors: Met52 treatment, spider treatment, spider survival at the end of experiment, and observation of the spider alive at the time of the final activity assessment. We used AICc values to compare the fits of alternative models.

## 3 RESULTS

### 3.1 Effects of Met52 and spiders on tick survival

#### 3.1.1 Flat nymph survival

**Figure 3:**
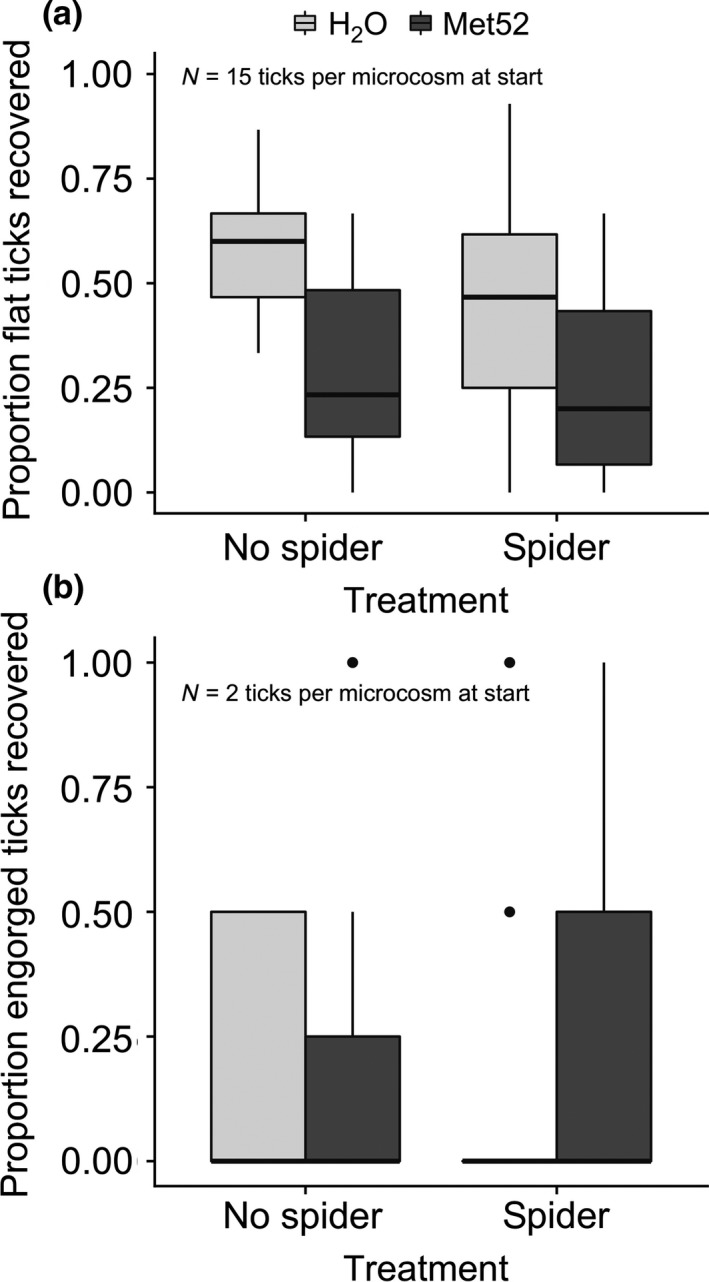
Boxplot of the proportion of *Ixodes scapularis* (a) flat nymphs and (b) engorged nymphs which survived and were recovered after 21 d in the field microcosms. There were a total of 88 microcosms in four treatments: no spider, H_2_O spray (*N* = 21 microcosms); spider addition, H_2_O spray (*N* = 20); no spider, Met52 spray (*N* = 24); and spider addition, Met52 spray (*N* = 23). Each microcosm initially had fifteen flat nymphs and two engorged nymphs. Boxes extend the interquartile range (IQR) from 25th to 75th percentile, and whiskers from IQR to 1.5*IQR, outliers are plotted individually. The best candidate model to explain survival of flat nymphal ticks included effects of both Met52 and spiders (Table 2), while the best candidate model to explain variation in the survival of engorged nymphs was the null (intercept‐only) model

Survival of unfed (flat) nymphal ticks was affected by both treatments: entomopathogenic fungus Met52 and the wolf spider *Schizocosa ocreata*. The best candidate model included effects of both Met52 and spiders. Similar levels of support (ΔAIC < 2) were observed for the models that included the Met52*spider interaction and for the Met52‐only model (Table 1). The fraction of flat nymphs surviving at the end of the experiment was highest in the microcosms receiving no spider and water spray and lowest in the spiders receiving spider and Met52 (Figure 3a). The Met52*spider interaction did not have a significant effect on nymph survival (*p* = 0.2) (Table 2).

**Table 1:** Comparison of alternative models for the fraction of flat nymphs surviving to be recovered at the end of the microcosm experiment

Model	Residual df	Number parameters	AICc	∆ AICc	Likelihood	AIC weight
spider + Met52	85	3	−13.71	0	1	0.48
spider + Met52 + spider*Met52	84	4	−13.06	0.65	0.72	0.35
Met52	86	2	−11.73	1.99	0.37	0.18
spider	86	2	7.86	21.57	0	0
intercept	87	1	8.95	22.66	0	0

**Table 2:** Summary of the fitted model including effects on flat nymph survival of Met52, wolf spider *S. ocreata*, and Met52*spider interaction. Met52 and spider addition each had significant negative effects on tick survival

Model	Coefficient estimate	Coefficient *SE*	*t* Value	*p*(>|*t*|)
Met52	−0.29	0.06	−4.55	<0.001
Spider	−0.16	0.07	−2.3	0.02
Met52*spider	0.11	0.09	1.24	0.2
Intercept	0.6	0.05	12.68	<0.001

#### 3.1.2 Engorged nymph survival

The best candidate model to explain variation in the survival of engorged nymphs was the null (intercept‐only) model, with similar levels of support for the spider model and the Met52 model (Table 3). Recovery of engorged nymphs was low overall, with a mode of 0, suggesting low statistical power to detect treatment effects (Figure 3b). There was a possible pattern of reduced survival in the microcosms receiving a spider and sprayed with H_2_O, compared to other treatments (Figure 3b).

**Table 3:** Comparison of alternative models for the fraction of engorged nymphs surviving and recovered at the end of the microcosm experiment

Model	Residual *df*	Number parameters	AICc	∆ AICc	Likelihood	AIC weight
Intercept	86	1	55.86	0	1	0.46
Spider	85	2	57.43	1.57	0.46	0.21
Met52	85	2	57.52	1.67	0.43	0.2
spider + Met52	84	3	59.16	3.3	0.19	0.09
spider + Met52 + spider*Met52	83	4	61.01	5.15	0.08	0.04

### 3.2 Effects of Met52 on spider survival

The best candidate model included an effect of Met52 on spider survival (Table 4). There was a significant negative effect of Met52 on spider survival (*z*_[1,41]_ = −2.774, *p* = 0.00554). In the water (control) microcosms, 70% (*SE* = 16%) of spiders survived, compared to 26% (*SE* = 5%) of spiders in the Met52 microcosms.

**Table 4:** Comparison of alternative models for spider survival to the end of the experiment

Model	Residual *df*	Number parameters	AICc	∆ AICc	Likelihood	AIC weight
Met52	41	2	55.14	0	1	0.96
intercept	42	1	61.5	6.36	0.04	0.04

### 3.3 Effects of Met52 and spiders on tick behavior

**Figure 4:**
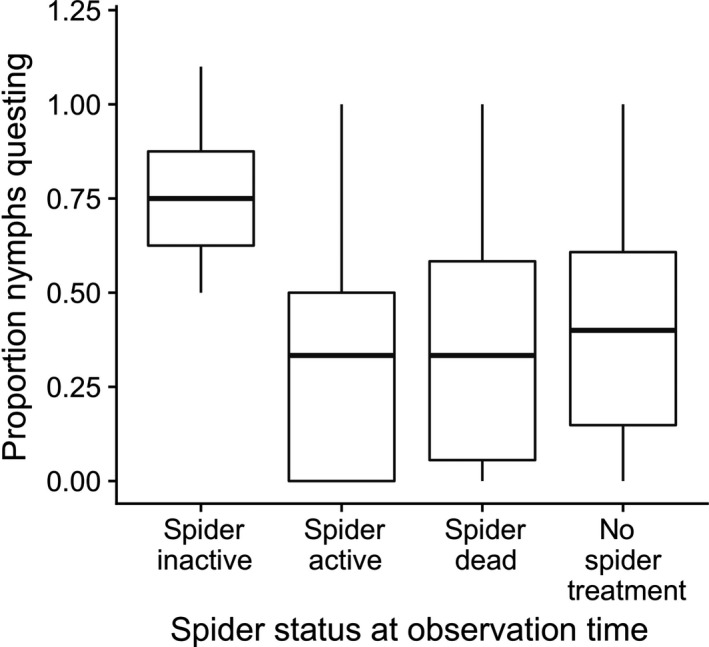
Boxplot of the proportion of flat nymphs of *Ixodes scapularis* seen in each microcosm immediately before removing them from the field, relative to the number of flat nymphs recovered from each microcosm at the end of the experiment. The categories are spider activity (spider active vs. not active during final observation), spider survival (spider alive at end of experiment vs. spider dead at end of experiment), and spider treatment (no spider vs. spider addition). There was a significant effect of spider activity on nymph questing. Boxes extend the interquartile range (IQR) from 25th to 75th percentile, and whiskers from IQR to 1.5*IQR, outliers are plotted individually

The best supported models for tick questing activity included effects of spider treatment, spider activity (seen vs. not seen in the final activity assessment) and spider survival to the end of the experiment (Table 5). In the model with the lowest AIC, there was a significant effect of spider activity, but no significant effect of spider treatment, or of whether the spider lived to the end of the experiment or not (Table 6). Ticks were more likely to quest in microcosms with a spider that lived to the end of the experiment but that was not active at the time of the final behavioral observation, compared to ticks in microcosms where we saw the spider active in the microcosm (Figure 4). The AICc values do not support Met52 affecting tick questing behavior.

**Table 5:** Comparison of alternative models for the number of nymphs observed questing in each microcosm immediately before the end of the experiment, as a fraction of the number of flat nymphs that survived to the end of the experiment

Model	Residual *df*	Number parameters	AICc	∆ AIC	Likelihood	AIC weight
spider treatment + spider active + spider lived	79	4	88	0	1	0.31
spider treatment + spider active	80	3	88.92	0.92	0.63	0.2
spider active + spider lived	80	3	88.94	0.94	0.62	0.19
Met52 + spider treatment + spider active + spider lived	78	5	90.2	2.2	0.33	0.1
Met52 + spider treatment + spider active	79	4	90.55	2.55	0.28	0.09
spider active	81	2	92.98	4.98	0.08	0.03
Intercept	82	1	93.22	5.22	0.07	0.02
spider treatment	81	2	93.64	5.64	0.06	0.02
Met52 + spider active	80	3	95.01	7.01	0.03	0.01
Met52	81	2	95.37	7.37	0.03	0.01
Met52 + spider treatment	80	3	95.84	7.84	0.02	0.01
spider survive	80	3	95.85	7.85	0.02	0.01
spider treatment + spider lived	80	3	95.85	7.85	0.02	0.01
Met52 + spider lived	80	3	97.04	9.04	0.01	0
Met52 + spider treatment + spider lived	79	4	98.1	10.1	0.01	0

**Table 6:** Summary of the fitted model of the proportion of questing nymphs, relative to the number of nymphs that survived, including effects of spider treatment, spider survival, and spider activity at time of observation. There was a significant effect of spider activity

Term	Coefficient estimate	Coefficient *SE*	*t* Value	*p*(>|*t*|)
spider active	−0.49	0.15	−3.2	0.002
spider treatment	0.19	0.11	1.76	0.081
spider lived	0.26	0.15	1.76	0.083
Intercept	0.41	0.06	6.76	0

## 4 DISCUSSION

*Metarhizium brunneum* strain F52 (Met52) and the brush‐legged wolf spider *Schizocosa ocreata* each reduced survival of *Ixodes scapularis,* consistent with previous studies (Bharadwaj & Stafford, 2010; Burtis & Pflueger, 2017; Hornbostel, Ostfeld, & Benjamin, 2005). In the context of this experiment, we considered the fungus and the wolf spider to have similar roles and to be of the same guild, while recognizing the differences between the two species (Simberloff & Dayan, 2008). We therefore consider the increased *S. ocreata* mortality in the Met52 microcosms to be evidence of intraguild predation. As *M. brunneum* infected *I. scapularis* and other prey, this may have facilitated exposure of *S. ocreata* to the fungus when *S. ocreata* attacked *I. scapularis* and other prey.

Despite the spider mortality caused by Met52, we did not detect a positive Met52*spider interaction effect on tick survival. The lack of risk reduction for ticks may be explained by the intraguild interaction being unidirectional, with the fungus killing the spider but the spider causing no interference to the fungus (Meyling & Hajek, 2010). When these two agents are deployed simultaneously, the effect of intraguild predation will reduce the impact of wolf spiders as a natural enemy of ticks, but this reduction may be outweighed by the relatively high efficacy of Met52 against ticks. The interference observed in this study will require further testing in residential yards, where the patterns of spatial overlap in microhabitats between Met52, *S. ocreata*, and *I. scapularis* may differ from the microcosms.

Our results suggest a need to further investigate the relative impacts of these biocontrol agents on different *I. scapularis* life stages. While Met52 and *S. ocreata* each effectively reduced flat nymphs, wolf spiders appeared to have a stronger effect than Met52 on the survival of engorged nymphs, based on the lowest recovery rate of engorged nymphs being in the microcosms receiving a spider and water spray (Figure 3). This result was not statistically significant, possibly due to low recovery rates, but it is consistent with previous observations that arthropod predators target engorged ticks more readily than flat ticks (Burtis & Pflueger, 2017; Samish & Alekseev, 2001). The sublethal effects of *M. brunneum* on *I. scapularis* have also been shown to vary by life stage (Hornbostel, Ostfeld, Zhioua, & Benjamin, 2004). These life stage dependent effects require more investigation, and suggest that accounting for the phenology of *I. scapularis,* relative to the phenology of natural enemies, has the potential to reduce interference between native and commercial biological control agents.

In addition to the direct effect of the wolf spider treatment on tick survival, we found a nonconsumptive effect of the spider on tick questing behavior. Ticks were more likely to quest if the spider was inactive and therefore unobserved at the time we made the questing assessment, compared to ticks in microcosms where the spider was active. This pattern of tick behavior is consistent with ticks undertaking risky questing behavior when spiders were less active. Web of Science searches (for “Ixod* AND preda*”; “Ixod* AND prey”; “Ixod* and “trait‐mediated”; Ixod* AND “nonconsumptive”) returned no prior studies reporting antipredator behavior in ticks. Further experiments would enable testing whether chemotactile cues from *S. ocreata* influence questing behavior or fitness of ticks (Schmitz, Miller, Trainor, & Abrahms, 2017). Chemotactile cues provide a possible mechanism by which ticks may modify their questing behavior in response to *S. ocreata*. Other taxa, for example, crickets *Gryllus pennsylvanicus,* avoid wolf spiders based on chemical cues (Storm & Lima, 2008). While *I. scapularis* response to chemical cues of arthropod predators has not yet been investigated, *I. scapularis* do respond to chemical cues of conspecifics (Allan & Sonenshine, 2003) and hosts (Carroll, Klun, & Schmidtmann, 1995). Cues from arthropod predators that influence tick questing behavior could influence contact rates between ticks and vertebrate hosts or people.

## 5 CONCLUSIONS

The biopesticide Met52 and the brush‐legged wolf spider *Schizocosa ocreata* each reduced the survival of flat *Ixodes scapularis* nymphs in field microcosms. Met52 also reduced survival of *S. ocreata*. The combination of Met52 and *S. ocreata* did not improve tick control. *I. scapularis* nymphs quested more when the spider in their microcosm was less active, suggesting that *I. scapularis* modified their behavior to reduce predation danger.

## CONFLICT OF INTEREST

None declared.

## AUTHORS’ CONTRIBUTIONS

All authors conceived the study; I.R.F. collected and analyzed the data and led the writing of the manuscript with the important contributions of J.C.B., F.K., and R.S.O.

## DATA ACCESSIBILITY

A data file is available from figshare https://figshare.com/s/dbe9cdc6a919f276dda7 (Fischhoff, Burtis et al. 2017)**.**
