# Supplementary material for: Tritrophic interactions between a fungal pathogen, a spider predator, and the blacklegged tick
Source: Ecol Evol. 2018 Jul 13;8(16):7824–34. doi: 10.1002/ece3.4271 (PMC6144966; doi:10.1002/ece3.4271)
Supplement: Supplementary file 1 [file ECE3-8-7824-s001.pdf]

Appendix S1: Table S1. Comparison of alternative models for the fraction of flat nymphs surviving to be recovered at the end of the microcosm experiment. The "gravid" model includes information about the female's reproductive status (with or without egg sacs), whereas the "spider" model does not include a reproductive status term.

Model	Residual df	Number parameters	AICc	Δ AIC	Likelihood	AIC weight
spider	86	2	7.86	0	1	0.55
gravid	85	3	8.29	0.43	0.81	0.45
